# Myeloma cells with asurophilic granules – an unusual morphological variant – case presentation

**Published:** 2008-02-25

**Authors:** AM Vlădăreanu, D Cîşleanu, M Derveşteanu, M Onisai, Horia Bumbea, S Radeşi, M Begu, C Băluţă

**Affiliations:** University of Medicine and Pharmacy Carol Davila – Clinic of Hematology Universitary Emergency Hospital BucharestRomania

**Keywords:** plasma cells, asurophilic granules, Russel bodies, Dutcher bodies

## Abstract

We present the case of an 80–year–old man who was admitted for anemia, back pain and progressive weakness. After a workup of clinical and laboratory data, the final diagnosis was multiple myeloma. The bone marrow aspirate revealed 53% myeloma cells with peculiar and rare  morphological features: numerous large asurophilic–bright red granules – mucopolizaccharides and immunoglobulins secreted and  accumulated in the endoplasmic reticulum, typically known as Russel bodies.

## Introduction

Multiple myeloma is a neoplastic plasma cell dyscrasia characterized by monoclonal proliferation of plasma cells with their accumulation in the bone marrow [1]. The malignant plasma cells produce an immunoglobulin (Ig) or protein M, a homogeneous Ig, formed of only one type of heavy chain and one type of light chain (kappa or lambda); the immunohistochemical particularities of this Ig establish the clinical features of the disease. 

Diagnostic criteria for multiple myeloma include: more than 10% atypical plasma cells in the bone marrow, a monoclonal immunoglobulin in the serum or light chains in the urine and the presence of osteolytic lesions [2].

Anemia, renal insufficiency, hypercalcemia, metabolic dysfunctions and infections are all clinical features of the disease, with prognostic value [3,4]. 

### Myeloma cells have different morphological variants

Usually, bone marrow aspirate shows clusters with a variable number of plasma cells; this underlines the need of careful examination of more than one smear. The bone marrow involvement is usually ‘focal’. Solitary plasma cell infiltration appears in rare cases [2].

Myeloma cells have distinct morphological criteria: [2, 5,6]

Myeloma cells are larger than reactive plasma cells, with high nucleo/cytoplasmic ratio. The nucleus is displaced from the center of the cell, with nodular chromatin pattern; some of the cells may present a nucleolus and a perinuclear clear zone. Multi – or binucleated plasma cells are present. The cytoplasm is basophilic with large intracytoplasmic inclusions (mucopolysaccharides and immunoglobulins secreted and accumulated in the endoplasmic reticulum), known as Russell bodies, resembling a bunch of grapes [2, 7]. Dutcher bodies are PAS positive intranuclear inclusions seen in plasma cells. In myeloma, there is often discordance between nucleus and cytoplasm, the former appearing immature and the latter highly differentiated. There is often a high polymorphism – there are seen both abnormal plasma cells, and transitional forms – lymphoplasmacytoid – cells with intermediate features between lymphocytes and plasma cells [2]. 

Disorders to be considered in the differential diagnosis of multiple myeloma include monoclonal gammopathy of undetermined significance (MGUS), smoldering myeloma, plasma cell leukemia, bone solitary plasmacytoma, extramedullar plasmacytoma, primary amyloidosis, chronic  lymphocytic leukemia and bone marrow metastasis [1].

The median survival period after diagnosis is approximately three years. Among the prognostic factors identified in this disease are: plasma cell proliferation index, β2 microglobulin level, C reactive protein, creatinine, lactate dehydrogenase (LDH), low serum albumin, plasmablast morphology [8], abnormal karyotypes (the complete deletion of chromosome 13) or t (4; 14), t (14;16) translocation [1,4]. Although there is virtually no prospect of a cure with current therapies (VMCP, VAD regimens etc, plus bisphosphonates) [[Bibr R9]–13], there are several options under investigation that include combinations of drugs such as Thalidomide, Bortezomib and CC – 5013 – Lenalidomide. [12, 14, 15] Several groups have used VAD followed by intensive myeloablative therapy with autologous marrow transplantation in younger patients [12, 16].

##  Full case presentation

We present the case of an 80 – year – old man, P.N. with percutaneous vertebroplasty in February 2007; he was in his usual state of health until two months prior to presentation, when he began to complain of back pain and progressive weakness.	

A complete blood cell count (CBC) revealed normochromic, normocytic anemia (hemoglobin 7.1 g/dl), rouleaux formation was appreciated on peripheral blood smear, biological inflammatory syndrome (ESR 60mm/hour, C reactive protein – positive), high lactate dehydrogenase (LDH 301U/l), creatinine 3.97mg/dl, BUN 157mg/dl, hypokaliemia (K 3.6mmols/l), hypercalcemia (Ca – 10.31mg/dl), hyperuricemia (8mg/dl). Serum protein electrophoresis detected a monoclonal peak limited to the gamma region of the electrophoretic strip (albumin 30%, alpha1 = 2.9%, alpha2 = 14.4%; beta = 8.7%; gamma = 44%); immunoelectrophoresis showed high values of IgG (35g/l), with low IgA (0.295g/l) and IgM (0.497g/l); urine immunofixation detected Bence Jones proteinuria. 

**Fig 1:**
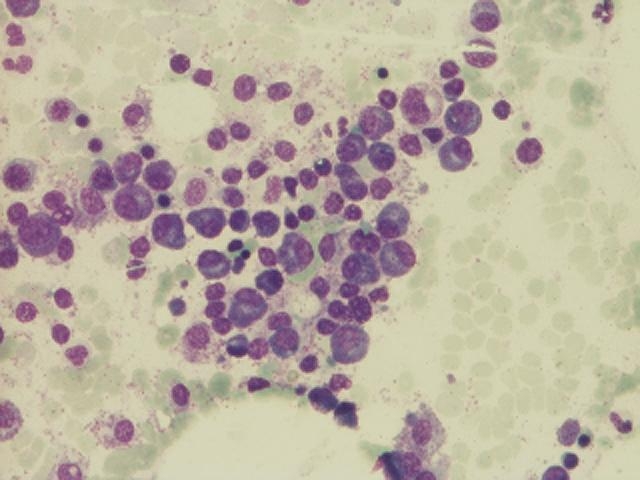
Clusters of plasma cells can be seen throughout this view of the aspirate – P.N. case (BM –MGG, objective 20X)

	Bone marrow aspiration was performed and revealed a hypercellular bone marrow with increased plasma cells (53%) with decreased granulocytic and erythrocytic components, with a slight megaloblastic deviation, and with present thrombocytogenic megakaryocytes (Fig 1)

**Fig 2:**
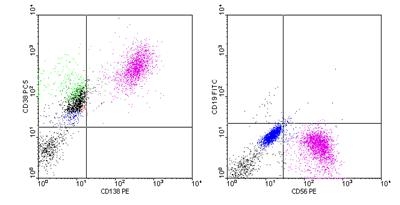
Dot–plot histograms: plasma cells (purple) positive for CD 38, CD 138, CD 56 and negative for CD 19 (BD Facs–Calibur, Software CellQuest version 3.3) –  P.N. case.

**Fig 3:**
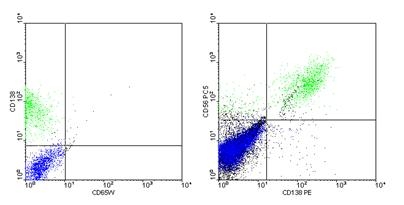
Dot –  plot histograms: plasma cells (purple) positive for CD 38, CD 138, CD 56 and negative for CD 19 (BD Facs –  Calibur, Software CellQuest version 3.3) –  P.N. case.

Flow cytometry analysis of the bone marrow aspirate is not included in the diagnostic criteria of multiple myeloma; in this case, no aberrant phenotype was noted - the profile of plasma cells was CD 38+ CD 138+ CD 56+(favorable prognostic factor) and CD 19 - (Fig 2,Fig 3).

**Fig 4:**
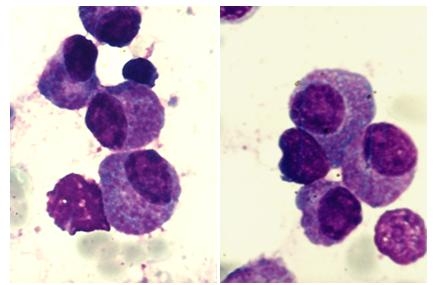
Clusters of plasma cells with azurophilic granules (MO – MGG, 100x objective) –P.N. case

The particular aspect of this case resides in the morphological features of the myeloma plasma cells (Fig 4 to Fig 7). These are large cells arranged in clusters, with basophilic cytoplasm, with excentrically placed nucleus and a perinuclear clear zone. The chromatin has a particular nodular pattern; an inconstant nucleolus may be observed. Plasma cells had numerous large azurophilic – bright red granules – mucopolysaccharides and immunoglobulins secreted and accumulated in the endoplasmic reticulum, typically known as Russel bodies [2, 5, 6]. 

**Fig 5:**
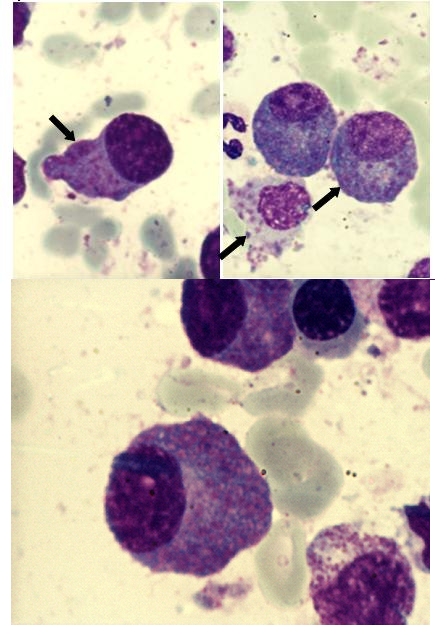
Plasma cells with numerous azurophilic granules that tend to cover the nucleus, in some cases with cohesive appearance (Auer rods–like) – P.N. case

**Fig 6:**
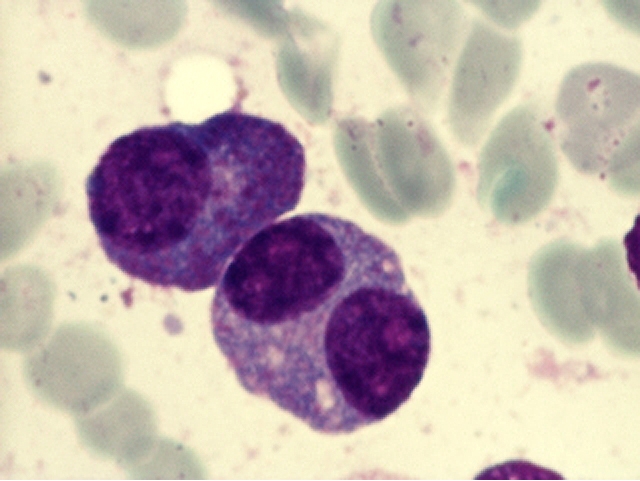
Plasma cells with numerous azurophilic granules, one binucleated, some granules have foamy aspect (MO – MGG, 100x objective) – P.N. case

**Fig 7:**
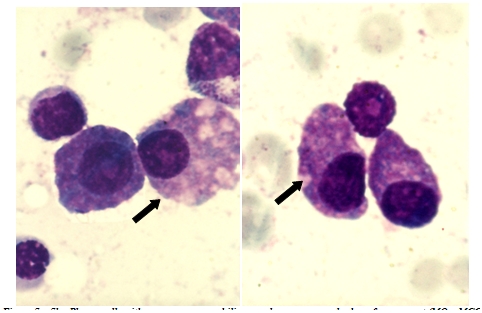
Plasma cells with numerous azurophilic granules, some granules have foamy aspect (MO – MGG, 100x objective) – P.N. case

**Fig 8:**
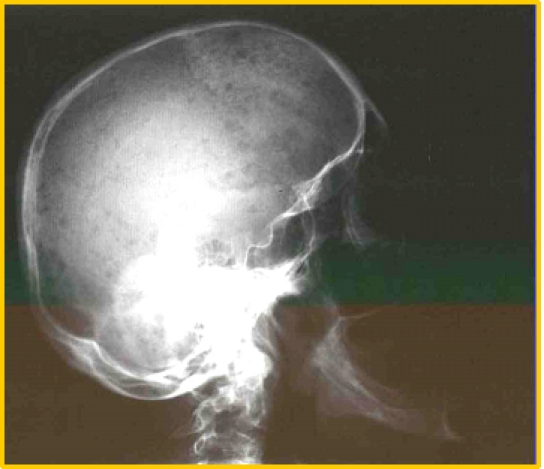
Skull x – ray showing multiple lytic areas – P.N. case

**Fig 9:**
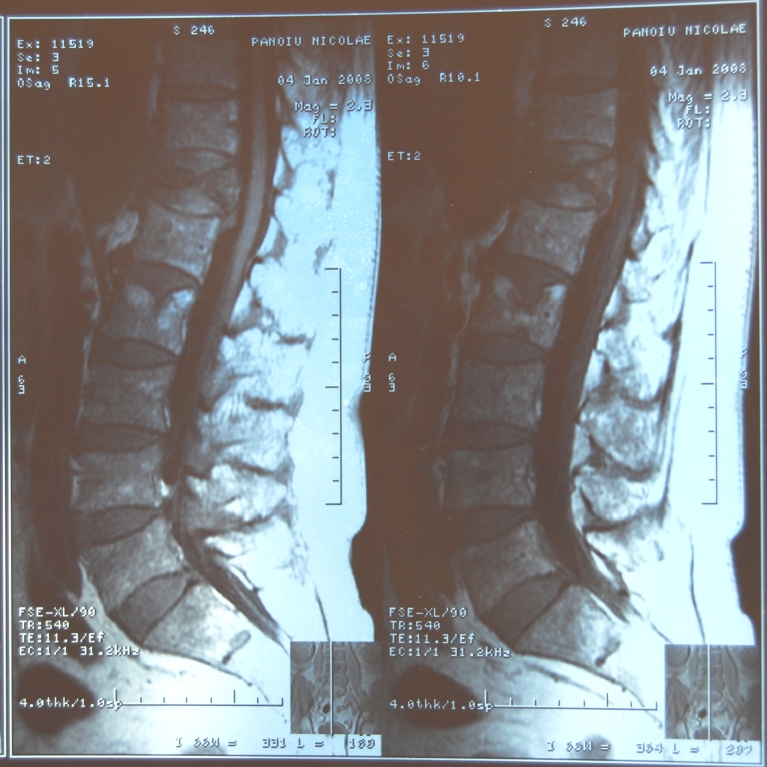
Spinal MRI scan – compression fracture of T12, L1, L2 with collapsed plateau – P.N. case

The investigations were completed with X – rays of the skull which showed multiple lytic cranial lesions and diffuse osteoporosis (Fig 8). Spinal MRI scan identified of compression fracture of T12, L1, L2 with diffuse osteoporosis (Fig 9).

The final diagnosis was Multiple Myeloma IgG Kappa stage III B; this was established according to Salmon–Durie Staging System [17] in the presence of major criteria: 53% myeloma plasma cells in bone marrow, monoclonal protein peak in serum, osteolytic lesions, corroborated with: anemia, renal failure, hypercalcemia. The multiple myeloma was complicated with compression fracture, secondary anemia, and possibly secondary amyloidosis. The renal failure may have a combined etiology: amyloidosis, myeloma kidney, excess NSAIDs treatment for back pain. 

With an obvious diagnosis, the differential diagnosis was easy to establish [1, 2, 3], including: a) monoclonal gammapathy of undetermined significance (MGUS) and b) smoldering myeloma, both with monoclonal immunoglobulin in the serum less than 3g/dl, > 10% plasma cells in bone marrow, no evidence of myeloma end – organ damage – anemia, renal failure, hypercalcemia, lytic lesions; c) Waldenstrom's macroglobulinemia with IgM paraprotein peak and lymphoplasmacytic bone marrow infiltration [1]. 

The patient's prognosis is unfavorable [1, 4] because of multiple associated factors: age, aggressive onset of disease – with complications (acute renal failure, multiple lytic lesions, vertebral destructions that needed surgical intervention), associated cardiac pathology which limits the possible therapeutic regimens, other biological factors with prognostic value – severe anemia, increased LDH, positive PCR. We mention that neither the percentage of plasma cell involvement of the bone marrow, nor the morphological aspect of plasma cells represent prognostic factors – except for the case when plasmablasts are observed, or when plasma cells appear on the peripheral blood smear (PBS) [1, 4].

## Discussion

 Multiple morphological variants of plasma cells are described [2, 5, 6]

- Plasma cells with abundant eosinophilic cytoplasm because of the presence of immunoglobulins as bright red granules – Flame Cells, frequently associated with IgA myeloma; however, they can also be associated with other types of myeloma.
- Plasma cells with globular inclusions (Russell bodies) in their cytoplasm – Mott cells – resembling a bunch of grapes
- Plasmablast – large cells with a very large nucleus and prominent nucleolus (adverse prognostic factor)
- Multiple intracellular mitosis and abnormal plasma cells with large nucleus

Plasma cells have a great variety of intracytoplasmic or intranuclear inclusions [2, 5, 6, 7]: Russell bodies (mucopolysaccharides and immunoglobulins secreted and accumulated in the endoplasmic reticulum), immunoglobulin granules and large vacuoles. These different globular intracytoplasmic inclusions are often mistaken for Auer rods or liposarcoma cells, or even adenocarcinoma cells – the typical aspect is of signet ring cells [6]. 

**Fig 10:**
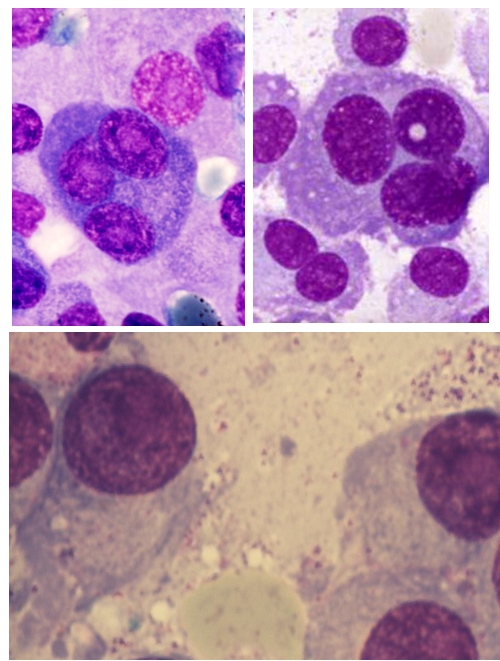
Typical plasma cells – cluster cells, eccentrically placed nucleus, nodular chromatin, basophilic cytoplasm, perinuclear clear zone, some multinucleated, others with nucleolus (B.M. – MGG, 100x objective) – images from ‘Diagnosis of Hematologic Malignancies – Atlas Notes and Images’, Ana Maria Vlădăreanu, Carol Davila Universitary Publishing House, Bucharest, 2007 – Hematology Department – Emergency Universitary Hospital Bucharest, with the author's permission

**Fig 11:**
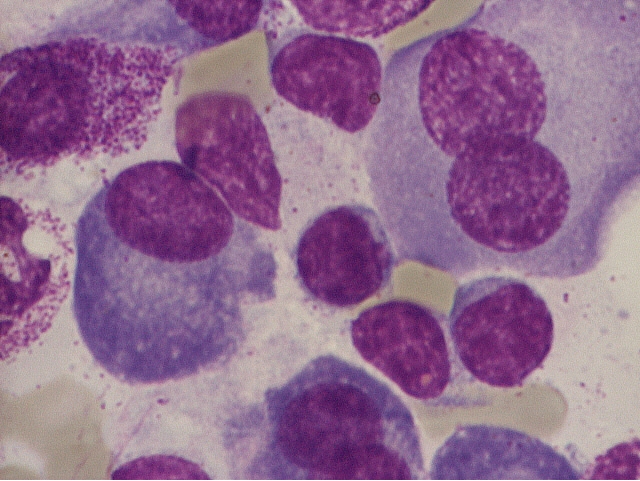
Typical plasma cells (nucleus displaced from the center, nodular chromatin, basophilic cytoplasm, a perinuclear clear zone, some multinucleated) and lymphoplasmacytoid cells (reduced basophilic cytoplasm, with high nucleo/cytoplasmic ratio) – (B.M. – MGG, 100x objective) – image from ‘Diagnosis of Hematologic Malignancies – Atlas Notes and Images’, Ana Maria Vlădăreanu, Carol Davila Universitary Publishing House, Bucharest, 2007 – Hematology Department – Emergency Universitary Hospital Bucharest, with the author's permission

**Fig 12:**
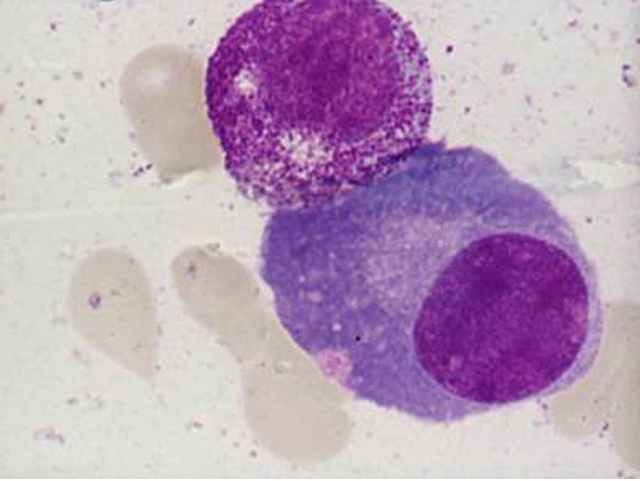
Plasma cells with Russell rods (B.M. – MGG, 100x objective) – image from ‘Diagnosis of Hematologic Malignancies – Atlas Notes and Images’, Ana Maria Vlădăreanu, Carol Davila Universitary Publishing House, Bucharest, 2007 – Hematology Department – Emergency Universitary Hospital Bucharest, with the author's permission

**Fig 13:**
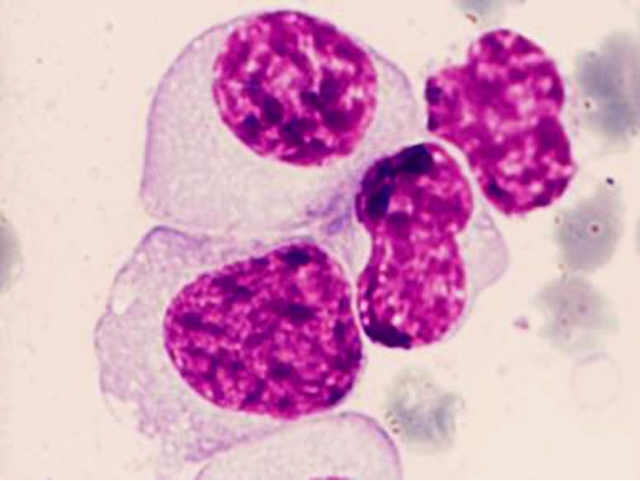
Plasma cell sarcoma like (B.M. – MGG, 100x objective) – image from ‘Diagnosis of Hematologic Malignancies – Atlas Notes and Images’, Ana Maria Vlădăreanu, Carol Davila Universitary Publishing House, Bucharest, 2007 – Hematology Department – Emergency Universitary Hospital Bucharest, with the author's permission

**Fig 14:**
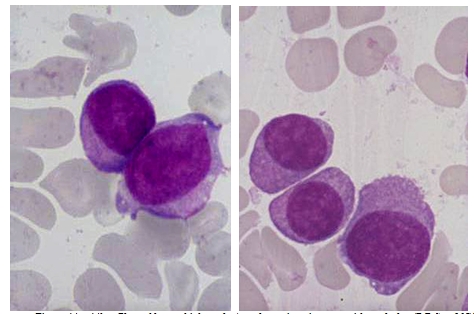
Plasmablasts – high nucleo/cytoplasmatic ratio, some with nucleolus (P.B.S. – MGG, 100x objective) – images from ‘Diagnosis of Hematologic Malignancies – Atlas Notes and Images’, Ana Maria Vlădăreanu, Carol Davila Universitary Publishing House, Bucharest, 2007 – Hematology Department – Emergency Universitary Hospital Bucharest, with the author's permission

**Fig 15:**
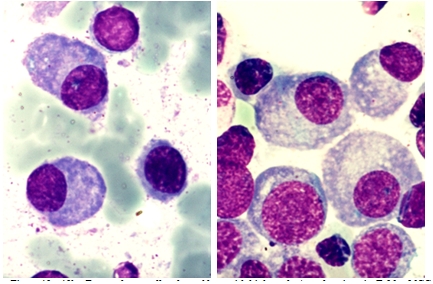
Foamy plasma cells, plasmablasts with high nucleo/cytoplasmic ratio (B.M. – MGG, 100x objective – images from the cases diagnosed in the Hematology Department – Emergency Universitary Hospital Bucharest)

**Fig 16:**
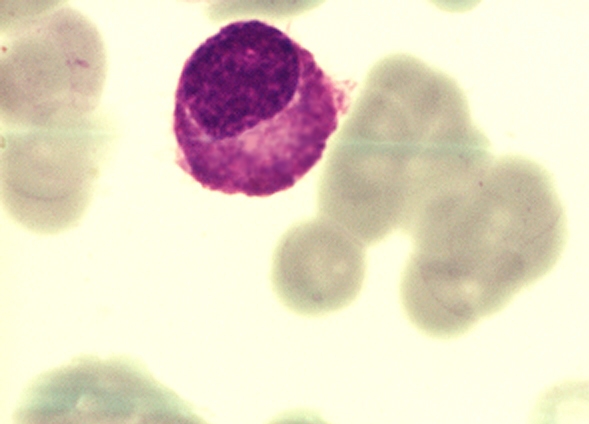
Flame plasma cell (B.M. – MGG, 100x objective – images from the cases diagnosed in the Hematology Department – Emergency Universitary Hospital Bucharest)

Because of the peculiar morphological aspect of plasma cells presented in this case (numerous large azurophilic – bright red granules), we considered necessary to have a careful differential diagnosis with other morphological variants [2, 5, 6, 7] – typical plasma cells (Fig 10, Fig 11), sarcoma plasma cells like (Fig 13), plasmablasts (Fig 14, Fig 15), foamy plasma cells (Fig 15), flame cells (Fig 16), Russell bodies (Fig 12) and reactive plasma cells. 

The morphological variants of plasma cells are presented below in Table 1:

**Table 1:** Different morphological aspects of plasma cells

*Type*	*Characteristics*
Plasma cells with or without anaplastic	high incidence
	nucleo/cytoplasmic asynchronism or dysplastic characteristics;
	nucleolus plasma cells
	anaplastic morphological type includes: large nucleus, polymorphism, morphological aspects resembling immunoblastic lymphoma
Small/lymphocytic cells	central nucleus, abundant or moderate basophilic cytoplasm;
	small incidence
Lobulated/ folded/monocytoid/ convoluted	small incidence
	lobulated nucleus and multiple irregularities
Flame Cells	polymorphic cells with eosinophilic cytoplasm (Flame Cells)
	torn cytoplasm cells
	multinucleated cells, associated with IgA myeloma, but also present in other myeloma types
Plasmablast	more than 2% blasts with high nucleo/cytoplasmic ratio, diffuse chromatin, variable nucleolus

There is a classification of multiple myeloma based on morphology, with important implications in evolution and prognosis

- Reduced malignancy MM: plasmocytic type
  - Small cells
- Intermediate malignancy MM: folded type
  - Polymorphous type
  - Asynchronic type
- High malignancy MM: plasmablastic type.

There are situations when it is necessary to make a differential diagnosis between plasma cells and blast cells in acute leukemia, and other hematopoietic precursors – osteoblast – or nonhematopoietic cells (bone marrow metastasis) [2, 5, 6,]

## Conclusions

The presented case illustrates the necessary diagnostic steps, a typical combination of clinical features and laboratory tests. 

It is a clear diagnosis: IgG Kappa multiple myeloma stage III B with compression fractures of T12, L1, L2, secondary anemia, possibly secondary amyloidosis and renal failure.

The unusual aspect of this case is the morphological facet of myeloma cells – with numerous large azurophilic–bright red granules; this characteristic, in the light of the multiple associated complications, may be regarded as an unfavorable prognostic factor in this particular case. 
